# Clinical effectiveness of mavacamten on cardiac function and disease-related indicators in patients with obstructive hypertrophic cardiomyopathy

**DOI:** 10.3389/fphar.2026.1861248

**Published:** 2026-10-27

**Authors:** Ya-Nan Shi, Jia-Jia Huang, Hong-Zhi Liu, Yu Xu, Fang Yuan

**Affiliations:** 1 Department of Pharmacy, Fuwai Hospital of Zhengzhou University, Heart Center of Henan Provincial People’s Hospital, Zhengzhou, Henan, China; 2 Department of Heart Failure, Fuwai Central China Cardiovascular Hospital, Central China Fuwai Hospital of Zhengzhou University, Heart Center of Henan Provincial People’s Hospital, Zhengzhou, Henan, China

**Keywords:** cardiac function, clinical effect, mavacamten, obstructive HCM, significant improvement

## Abstract

**Objective:**

This study aimed to compare the clinical effects of mavacamten and conventional pharmacotherapy on cardiac function and related disease indicators in patients with obstructive hypertrophic cardiomyopathy (oHCM).

**Methods:**

A retrospective analysis was performed in patients with oHCM who were hospitalized between January 2024 and May 2025. The intervention group included 126 patients who received mavacamten for the first time, while the control group consisted of 134 patients treated with conventional medications. Baseline clinical characteristics, laboratory parameters, and imaging findings were collected. All patients were followed up for 30 weeks after discharge. Changes in relevant clinical indicators were recorded and compared at weeks 6 (W6), 18 (W18), and 30 (W30).

**Results:**

A total of 222 patients (111 per group) completed the 30-week follow-up and were included in the final analysis. After 30 weeks of treatment, the resting left ventricular outflow tract (LVOT) peak gradient fell to 23.76 ± 4.09 mmHg in the intervention group but persisted at 78.49 ± 13.21 mmHg in the control group (t = 24.074, p < 0.001). Similarly, the Valsalva-provoked LVOT peak gradient decreased to 48.95 ± 11.10 mmHg versus 113.59 ± 14.92 mmHg in the control group (t = 21.147, p < 0.001). Left ventricular ejection fraction (LVEF) was 55.12% ± 4.85% in the intervention group and 56.78% ± 5.42% in the control group (p = 0.312), with 9.5% of mavacamten-treated patients experiencing LVEF decline to <50% (vs. 2.2% in controls). N-terminal pro-B-type natriuretic peptide (NT-proBNP) and high-sensitivity cardiac troponin I (hs-cTnI) levels were significantly lower in the intervention group (p < 0.05).

**Conclusion:**

Mavacamten indicates significant clinical association with improved outcomes in improving cardiac function and related disease indicators in patients with obstructive hypertrophic cardiomyopathy, with sustained reductions in both resting and provoked LVOT gradients.

## 1 Introduction

Hypertrophic cardiomyopathy (HCM) is a myocardial disorder characterized by primary left ventricular hypertrophy. Approximately two-thirds of affected individuals present with dynamic left ventricular outflow tract (LVOT) obstruction, a condition defined as obstructive hypertrophic cardiomyopathy (oHCM). With advances in genetic testing technologies, epidemiological data indicate that the estimated prevalence of HCM has increased from 0.16%–0.23% to 0.2%–0.5%, with pathogenic gene variants identified in approximately 60% of individuals with HCM (Maron et al., 2022; Pascoe, 2018; Chinese Medical Doctor Association, 2017).

Mavacamten is the first selective cardiac myosin inhibitor developed to directly target the underlying pathophysiological mechanism of oHCM (Schenk and Fields, 2023; Rader et al., 2024). The drug was approved for marketing in China on 24 April 2024, for the treatment of patients with obstructive hypertrophic cardiomyopathy classified as New York Heart Association (NYHA) functional class II–III, with the therapeutic goal of improving exercise capacity and clinical symptoms. This therapeutic strategy represents a transition from conventional symptomatic management to mechanism-based treatment (Toepfer et al., 2019; Anderson et al., 2018; Mamidi et al., 2018). Although the phase III EXPLORER-CN clinical trial has been conducted in China, post-marketing clinical data evaluating treatment benefits in Chinese patients remain limited. Therefore, the present study was designed to analyze the clinical effects of mavacamten on cardiac function and other disease-related indicators in patients with oHCM.

## 2 Study participants and methods

### 2.1 Study population

This retrospective analysis consecutively enrolled patients with oHCM hospitalized at Fuwai Central China Cardiovascular Hospital between January 2024 and May 2025. This retrospective observational study was approved by the Ethics Committee of Fuwai Central China Cardiovascular Hospital (Ethical 2022 No. 8), and the requirement for written informed consent was waived due to its retrospective design with de-identified clinical data analyzed anonymously. All procedures were performed in accordance with the Declaration of Helsinki and relevant clinical research guidelines.

A total of 317 patients were initially screened, among whom 57 were excluded based on predefined criteria: age <18 years (n = 19), NYHA functional class I/IV (n = 14), severe renal or hepatic dysfunction (n = 12), incomplete baseline echocardiographic data (n = 8), and refusal of follow-up (n = 4). The remaining 260 eligible patients were grouped according to mavacamten clinical availability (approved on 24 April 2024), standardized clinical eligibility, and comprehensive physician judgment. The intervention group (n = 126) received initial mavacamten treatment, whereas the control group (n = 134) initiated first-line conventional pharmacotherapy with beta-blockers or non-dihydropyridine calcium channel blockers. After a 30-week follow-up period, 38 patients were lost to follow-up (15 in the intervention group and 23 in the control group). The final analytical cohort comprised 222 patients, with 111 patients in each group (Supplementary Figure S1).

#### 2.1.1 Diagnostic criteria for oHCM

Consistent with the 2023 Chinese Guidelines for Diagnosis and Treatment of Hypertrophic Cardiomyopathy in Adults. Resting LVOT peak gradient ≥30 mmHg, or Valsalva-induced LVOT peak gradient ≥30 mmHg detected by transthoracic echocardiography, combined with myocardial hypertrophy (interventricular septal thickness ≥15 mm, or ≥13 mm with family history of HCM). Baseline Left ventricular ejection fraction (LVEF) inclusion threshold: Baseline LVEF ≥55% at initial admission echocardiography, consistent with domestic real-world clinical medication standards for mavacamten.

#### 2.1.2 Inclusion and exclusion criteria

All enrolled subjects met the core oHCM diagnostic criteria above, with the following uniform exclusion criteria:

(1) Age <18 years; (2) NYHA cardiac function class I or IV; (3) Baseline LVEF <55%; (4) Combined severe valvular heart disease, dilated cardiomyopathy, ischemic cardiomyopathy, or history of myocardial infarction; (5) Severe liver and renal insufficiency (serum creatinine >133 μmol/L, transaminase >3 times upper limit of normal); (6) Pregnancy or lactation; (7) Incomplete baseline imaging and laboratory data, unable to complete scheduled follow-up visits.

### 2.2 Treatment protocol

#### 2.2.1 Mavacamten dosing protocol

In the intervention group, mavacamten was administered in accordance with the domestic clinical protocol established by Fuwai Central China Cardiovascular Hospital, adapted from the EXPLORER-HCM and EXPLORER-CN trial protocols. The starting dose was 2.5 mg once daily. Dose titration was performed at W6, W12 and W18 based on serial echocardiographic assessments of LVEF and LVOT gradient. Up-titration to 5 mg, 10 mg, and subsequently to 15 mg once daily (maximum dose, Table 1) was permitted if the resting LVOT gradient remained ≥30 mmHg and LVEF was ≥55%. Dose reduction by one level was mandated if LVEF declined to 45%–54%. Mavacamten was discontinued immediately if LVEF fell below 45%, and the patient was withdrawn from the study intervention arm. To ensure medication administration adhered to standard clinical protocols, the following verification steps were performed: (1) Electronic prescription review: All mavacamten prescriptions were cross-checked against the hospital pharmacy dispensing records to confirm dose and duration; (2) Outpatient follow-up records: Dose adjustments at W6, W12, W18 and W24 were verified against echocardiography reports and physician notes; (3) Adverse event documentation: The 8 patients with LVEF decline had complete documentation of dose reduction/discontinuation per protocol.

**TABLE 1:** Mavacamten dose titration and resting LVOT gradient reduction.

Mavacamten dose	n	Baseline resting LVOT gradient (mmHg)	W30 resting LVOT gradient (mmHg)	Mean reduction (mmHg)	95% CI
2.5 mg	18	82.4 ± 12.1	38.6 ± 8.5	43.8	36.2–51.4
5 mg	32	80.1 ± 11.8	31.2 ± 7.3	48.9	42.1–55.7
10 mg	42	78.5 ± 10.9	24.8 ± 6.1	53.7	48.3–59.1
15 mg	19	76.2 ± 11.3	18.5 ± 4.2	57.7	51.4–64.0

#### 2.2.2 Control group treatment protocol

Patients in the control group received conventional pharmacotherapy as first-line treatment for symptomatic oHCM, prescribed at the discretion of the treating physician according to the 2024 AHA/ACC/AMSSM/HRS/PACES/SCMR Guideline (Ommen et al., 2024) and 2023 Chinese Guidelines for Diagnosis and Treatment of Hypertrophic Cardiomyopathy in Adults (National Center for Cardiovascular Diseases, 2023). The regimen comprised β-blockers (metoprolol succinate, bisoprolol, or carvedilol, titrated to maximally tolerated doses), non-dihydropyridine calcium channel blockers (verapamil or diltiazem, in patients without significant resting obstruction or conduction abnormalities), or a combination thereof. Dose adjustments were made at the same clinical visits as the intervention group (Week 4 and Week 12) based on heart rate, blood pressure, and symptomatic response. No patient in the control group received mavacamten during the 30-week observation period.

### 2.3 Observational indicators

Baseline clinical characteristics were retrospectively collected at enrolment, including demographics, NYHA functional class, comorbidities, vital signs, echocardiographic indices (LVEF, left ventricular end-diastolic diameter (LVEDD), left ventricular end-systolic diameter (LVESD), left atrial volume index (LAVI), right atrial transverse diameter (RATD)), and circulating biomarkers (N-terminal pro-B-type natriuretic peptide (NT-proBNP), high-sensitivity cardiac troponin I (hs-cTnI)). Tricuspid annular plane systolic excursion (TAPSE) was assessed as a secondary indicator of right ventricular function. Serial assessments including echocardiography, laboratory indicators, Kansas City Cardiomyopathy Questionnaire-23 Clinical Summary Score (KCCQ-23CSS) questionnaires and 6-min walk tests (6MWT) were performed repeatedly at baseline, Weeks 6, 18 and 30. Patients underwent routine outpatient clinical evaluations at Weeks 4 and 12 after treatment initiation for safety monitoring and dose titration.

For echocardiography, LVEF was quantified via the manual Simpson’s biplane method from apical four- and two-chamber views, with papillary muscles excluded during tracing. All images were interpreted by two independent senior echocardiographers blinded to treatment allocation, with an inter-observer intraclass correlation coefficient (ICC) of 0.94; any measurement discrepancies exceeding 5% were resolved by joint consensus reading. M-mode tracing from the apical four-chamber view was used to measure TAPSE. Echocardiographic findings guided dose titration at Weeks 6, 12, 18 and 24.

Blinded assessors administered the KCCQ-23CSS (five subdomains, scoring range 0–100) and standardized 6MWT following American Thoracic Society guidelines at all scheduled timepoints.

### 2.4 Statistical analysis

Statistical analyses were performed using R software version 4.3.3. The Shapiro–Wilk test was applied to assess data normality. Continuous variables with a normal distribution were expressed as mean ± standard deviation (*x̄* ± *s*) and were compared using the independent-samples *t*-test between groups. Continuous variables with a non-normal distribution were presented as median (interquartile range) [M (P25, P75)] and were compared using the Mann-Whitney U test. A two-sided *p* value <0.05 was considered statistically significant. This study was a retrospective observational study, and the patients were consecutively enrolled. The sample size was determined by the number of available cases during the study period without prospective sample size estimation. Post-hoc power analysis demonstrated that the existing sample size provided extremely high statistical power (>99.9%) for the primary endpoints (LVOT gradients); for some secondary endpoints (e.g., NT-proBNP), power was approximately 85%, with potential risk of Type II error. Primary longitudinal analyses were conducted using linear mixed-effects models (LMM) with restricted maximum likelihood estimation. The model included fixed effects for treatment group, time (Baseline, W6, W18, W30), and their interaction, with baseline LVEDD, LAVI, LVESV, age, sex, and baseline outcome value as covariates. Subject-specific random intercepts were included to account for within-subject correlation. First-order autoregressive [AR(1)] residual covariance structure was specified based on Akaike information criterion comparison (Table 2). For non-normally distributed outcomes (NT-proBNP), log-transformation was applied prior to LMM fitting. Sensitivity analyses included generalized estimating equations (GEE) with robust standard errors and pattern-mixture models for missing data. For transparency, the original independent-samples t-tests at each time point are retained as Supplementary Table S1, but all primary inferences are based on LMM results.

**TABLE 2:** Baseline characteristics before and after propensity score matching.

Variable	Before matching	After matching	SMD	Control (n = 111)	Before	After
	Intervention (n = 126)	Control (n = 134)	Intervention (n = 111)			
Age (years)	51.53 ± 13.49	57.21 ± 13.38	54.48 ± 13.20	52.61 ± 13.58	−0.423	0.140
Male sex (%)	52.4	53.7	60.4	57.7	−0.027	0.055
NYHA class III (%)	42.1	37.3	41.4	37.8	0.097	0.073
Resting LVOT gradient (mmHg)	79.27 ± 11.37	75.51 ± 11.80	77.99 ± 10.90	77.04 ± 10.90	0.325	0.088
LVEF (%)	59.17 ± 3.21	60.37 ± 3.15	59.57 ± 2.95	59.75 ± 2.88	−0.374	−0.073
NT-proBNP (ng/L)	4,488.94 ± 2,954.60	4,220.03 ± 3,297.77	4,252.10 ± 3,042.30	4,812.35 ± 3,300.62	0.086	−0.177
Left atrial diameter (mm)	41.24 ± 6.78	37.21 ± 4.57	39.32 ± 6.02	38.70 ± 6.08	0.697	0.102
Beta-blocker use (%)	59.5	79.1	64.9	74.8	−0.433	−0.216

## 3 Results

### 3.1 Demographic and clinical characteristics

A total of 222 patients (111 per group) who completed 30-week follow-up were included in the final analysis. Baseline characteristics were generally comparable between groups, with significant differences in LVEDD (51.61 ± 9.11 vs. 56.22 ± 6.57 mm, p = 0.015), LAVI (58.0 ± 19.8 vs. 47.8 ± 12.4 mL/m^2^, p = 0.008), and LVESV [45.00 (32.00, 51.00) vs. 50.00 (44.00, 56.00) mL, p = 0.045] (Table 3; Table 4). Heart rate remained stable within and between groups throughout follow-up (Table 5; Table 6).

**TABLE 3:** Baseline demographic and clinical characteristics of the two groups.

Variable	Intervention (*n* = 111)	Control (*n* = 111)	*t/z*	*p*
Heart rate (bpm)	73.68 ± 12.54	78.59 ± 11.15	1.783	0.079
SBP (mmHg)	125.35 ± 22.41	125.92 ± 15.44	0.127	0.899
DBP (mmHg)	80.73 ± 12.99	77.38 ± 7.99	1.337	0.186
LVEF (%)	58.42 ± 2.89	58.38 ± 2.68	0.488	0.626
LVEDD (mm)	51.61 ± 9.11	56.22 ± 6.57	2.493	0.015*
LVESD (mm)	32.56 ± 9.81	32.11 ± 3.46	0.262	0.794
LAVI (mL/m^2^)	58.0 ± 19.8	47.8 ± 12.4	2.731	0.008**
RATD (mm)	34.92 ± 3.83	34.97 ± 3.44	0.064	0.949
LVEDV (mL)	104.00[90.53,117.69]	107.00[94.00,118.00]	0.119	0.905
LVEDVi (mL/m^2^)	60.1 [52.3, 68.0]	61.8 [54.3, 68.2]	0.122	0.897
LVESV (mL)	45.00[32.00,51.00]	50.00[44.00,56.00]	2.006	0.045*
IVST (mm)	22.84 ± 3.51	22.45 ± 1.03	0.644	0.522
LVOT (mm)	12.79 ± 5.49	12.03 ± 1.40	0.802	0.427
LVOT gradient (resting) (mmHg)	79.24 ± 11.61	75.46 ± 11.86	1.387	0.170
LVOT gradient (provoked) (mmHg)	105.08 ± 15.90	102.68 ± 18.76	0.595	0.554
pvo2	24.97 ± 3.35	27.27 ± 13.35	1.016	0.316
Hs-cTnI	12.35 ± 2.21	13.34 ± 3.10	1.593	0.116
KCCQ-23CSS	58.35 ± 7.93	55.30 ± 7.08	1.747	0.085
Tapse	20.35 ± 5.92	23.59 ± 8.06	1.972	0.052
E/e	16.04 ± 6.20	17.35 ± 6.62	0.878	0.383
ST2	22.31 ± 7.32	19.92 ± 5.96	1.540	0.128
GAL-3	20.06 ± 5.86	18.22 ± 5.66	1.369	0.175
NT-proBNP (pg/mL)	3,611.30[2,482.93,5099.98]	3,595.73[1,610.92,4683.76]	0.816	0.414

**p < 0.01, *p < 0.05. Abbreviations: bpm, beats per minute; SBP, systolic blood pressure; DBP, diastolic blood pressure; LVEF, left ventricular ejection fraction; LVEDD, left ventricular end-diastolic diameter; LVESD, left ventricular end-systolic diameter; LAVI, left atrial volume index; RATD, right atrial transverse diameter; LVEDV, left ventricular end-diastolic volume; LVEDVi, left ventricular end-diastolic volume index; LVESV, left ventricular end-systolic volume; IVST, interventricular septal thickness; LVOT, left ventricular outflow tract; pvo_2_, partial pressure of venous oxygen; hs-cTnI, high-sensitivity cardiac troponin I; KCCQ-23CSS, Kansas City Cardiomyopathy Questionnaire-23 clinical summary score; TAPSE, tricuspid annular plane systolic excursion; E/e, ratio of early transmitral flow velocity to early mitral annular tissue velocity; ST2, suppression of tumorigenicity 2; GAL-3, galectin-3; NT-proBNP, N-terminal pro-B-type natriuretic peptide.

**TABLE 4:** Adjusted treatment effects on primary and secondary outcomes.

Endpoint	Timepoint	Analysis	Adjusted between-group difference (95% CI)	P-value
Resting LVOT gradient (mmHg)	Week 6	Unadjusted	−30.15 (−33.42 to −26.88)	<0.001
Resting LVOT gradient (mmHg)	Week 6	Adjusted for LVEDD, LAVI, LVESV	−28.82 (−32.31 to −25.33)	<0.001
Resting LVOT gradient (mmHg)	Week 6	Fully adjusted*	−28.42 (−32.08 to −24.76)	<0.001
Resting LVOT gradient (mmHg)	Week 18	Unadjusted	−45.30 (−49.12 to −41.48)	<0.001
Resting LVOT gradient (mmHg)	Week 18	Adjusted for LVEDD, LAVI, LVESV	−43.15 (−47.24 to −39.06)	<0.001
Resting LVOT gradient (mmHg)	Week 18	Fully adjusted*	−42.58 (−46.82 to −38.34)	<0.001
Resting LVOT gradient (mmHg)	Week 30	Unadjusted	−54.73 (−57.89 to −51.57)	<0.001
Resting LVOT gradient (mmHg)	Week 30	Adjusted for LVEDD, LAVI, LVESV	−52.18 (−55.62 to −48.74)	<0.001
Resting LVOT gradient (mmHg)	Week 30	Fully adjusted*	−50.96 (−54.71 to −47.21)	<0.001
Valsalva LVOT gradient (mmHg)	Week 6	Unadjusted	−38.40 (−42.15 to −34.65)	<0.001
Valsalva LVOT gradient (mmHg)	Week 6	Adjusted for LVEDD, LAVI, LVESV	−36.55 (−40.52 to −32.58)	<0.001
Valsalva LVOT gradient (mmHg)	Week 6	Fully adjusted*	−35.18 (−39.48 to −30.88)	<0.001
Valsalva LVOT gradient (mmHg)	Week 18	Unadjusted	−55.60 (−59.85 to −51.35)	<0.001
Valsalva LVOT gradient (mmHg)	Week 18	Adjusted for LVEDD, LAVI, LVESV	−53.20 (−57.72 to −48.68)	<0.001
Valsalva LVOT gradient (mmHg)	Week 18	Fully adjusted*	−52.75 (−57.38 to −48.12)	<0.001
Valsalva LVOT gradient (mmHg)	Week 30	Unadjusted	−64.64 (−68.31 to −60.97)	<0.001
Valsalva LVOT gradient (mmHg)	Week 30	Adjusted for LVEDD, LAVI, LVESV	−61.85 (−65.82 to −57.88)	<0.001
Valsalva LVOT gradient (mmHg)	Week 30	Fully adjusted*	−60.42 (−64.68 to −56.16)	<0.001
NT-proBNP (ng/L)	Week 6	Unadjusted	−450.00 (−1,280.00–380.00)	0.285
NT-proBNP (ng/L)	Week 6	Adjusted for LVEDD, LAVI, LVESV	−380.00 (−1,150.00–390.00)	0.332
NT-proBNP (ng/L)	Week 6	Fully adjusted*	−320.00 (−1,080.00–440.00)	0.408
NT-proBNP (ng/L)	Week 18	Unadjusted	−980.00 (−2,150.00–190.00)	0.089
NT-proBNP (ng/L)	Week 18	Adjusted for LVEDD, LAVI, LVESV	−1,150.00 (−2,380.00 to 80.00)	0.052
NT-proBNP (ng/L)	Week 18	Fully adjusted*	−1,050.00 (−2,280.00 to 180.00)	0.078
NT-proBNP (ng/L)	Week 30	Unadjusted	−1,280.00 (−2,480.00 to −80.00)	0.040**
NT-proBNP (ng/L)	Week 30	Adjusted for LVEDD, LAVI, LVESV	−1,150.00 (−2,380.00 to 80.00)	0.052
NT-proBNP (ng/L)	Week 30	Fully adjusted*	−980.00 (−2,210.00–250.00)	0.098
Hs-cTnI (ng/L)	Week 6	Unadjusted	−4.52 (−6.12 to −2.92)	<0.001
Hs-cTnI (ng/L)	Week 6	Adjusted for LVEDD, LAVI, LVESV	−4.28 (−5.98 to −2.58)	<0.001
Hs-cTnI (ng/L)	Week 6	Fully adjusted*	−4.15 (−5.82 to −2.48)	<0.001
Hs-cTnI (ng/L)	Week 18	Unadjusted	−5.85 (−7.52 to −4.18)	<0.001
Hs-cTnI (ng/L)	Week 18	Adjusted for LVEDD, LAVI, LVESV	−5.55 (−7.32 to −3.78)	<0.001
Hs-cTnI (ng/L)	Week 18	Fully adjusted*	−5.48 (−7.18 to −3.78)	<0.001
Hs-cTnI (ng/L)	Week 30	Unadjusted	−6.69 (−8.12 to −5.26)	<0.001
Hs-cTnI (ng/L)	Week 30	Adjusted for LVEDD, LAVI, LVESV	−6.31 (−7.82 to −4.80)	<0.001
Hs-cTnI (ng/L)	Week 30	Fully adjusted*	−5.98 (−7.65 to −4.31)	<0.001
LVEF (%)	Week 6	Unadjusted	−2.85 (−5.92 to 0.22)	0.068
LVEF (%)	Week 6	Adjusted for LVEDD, LAVI, LVESV	−2.62 (−5.78 to 0.54)	0.102
LVEF (%)	Week 6	Fully adjusted*	−2.45 (−5.68 to 0.78)	0.135
LVEF (%)	Week 18	Unadjusted	−4.80 (−8.55 to −1.05)	0.012*
LVEF (%)	Week 18	Adjusted for LVEDD, LAVI, LVESV	−4.52 (−8.38 to −0.66)	0.022*
LVEF (%)	Week 18	Fully adjusted*	−4.15 (−8.12 to −0.18)	0.041*
LVEF (%)	Week 30	Unadjusted	−6.41 (−10.82 to −2.00)	0.007**
LVEF (%)	Week 30	Adjusted for LVEDD, LAVI, LVESV	−5.89 (−10.45 to −1.33)	0.012*
LVEF (%)	Week 30	Fully adjusted*	−5.52 (−10.28 to −0.76)	0.024*

*Fully adjusted model includes baseline LVEDD, LAVI, LVESV, age, sex, and baseline outcome value as covariates. *P < 0.05; **P < 0.01.

**TABLE 5:** Heart rate at each follow-up time point.

Time point	Intervention group (n = 111)	Control group (n = 111)	t-value	P-value
Baseline	73.68 ± 12.54	78.59 ± 11.15	1.783	0.079
Week 6 (W6)	75.64 ± 11.55	77.45 ± 11.58	2.368	0.065
Week 18 (W18)	73.35 ± 12.08	74.67 ± 10.05	2.885	0.072
Week 30 (W30)	74.08 ± 12.25	76.38 ± 11.99	0.815	0.418

**TABLE 6:** Within-group comparison of heart rate (vs. Baseline).

Group	W6 vs. Baseline	W18 vs. Baseline	W30 vs. Baseline
Intervention	t = 1.030, P = 0.305	t = 0.555, P = 0.580	t = −1.666, P = 0.099
Control	t = 1.609, P = 0.110	t = 0.912, P = 0.364	t = 1.977, P = 0.051

Heart rate was systematically recorded at each transthoracic echocardiographic examination to account for its potential influence on LVOT pressure gradients. As shown in Table 5, Table 6, the intervention group demonstrated numerically lower heart rate values compared with the control group at all time points. However, no statistically significant between-group differences were observed at baseline (73.68 ± 12.54 vs. 78.59 ± 11.15 bpm, P = 0.079), W6 (75.64 ± 10.05 vs. 77.45 ± 11.58 bpm, P = 0.065), W18 (73.35 ± 12.08 vs. 74.67 ± 10.05 bpm, P = 0.072), or W30 (74.08 ± 12.25 vs. 76.38 ± 11.99 bpm, P = 0.418). Within-group analysis revealed that heart rate remained relatively stable throughout the follow-up period in both groups, with no statistically significant changes from baseline to W30 (intervention: P = 0.099; control: P = 0.051). These findings suggest that the observed reductions in LVOT gradients in the mavacamten group are unlikely to be primarily mediated by heart rate changes.

### 3.2 Primary and secondary outcomes at week 30

At W30, the intervention group demonstrated significantly lower resting LVOT gradient (23.76 ± 4.09 vs. 78.49 ± 13.21 mmHg), provoked LVOT gradient (48.95 ± 11.10 vs. 113.59 ± 14.92 mmHg), hs-cTnI (6.50 ± 1.41 vs. 13.19 ± 3.09 ng/L), and NT-proBNP [1,082.00 (661.00, 4,211.00) vs. 2,361.00 (1,227.00, 5,059.00) pg/mL] compared with the control group (all p < 0.05). TAPSE was higher in the intervention group (19.49 ± 7.77 vs. 16.19 ± 5.29 mm, p = 0.036), consistent with preserved right ventricular longitudinal function. LVEF did not differ significantly between groups (55.12% ± 4.85% vs. 56.78% ± 5.42%, p = 0.312). KCCQ-23CSS was comparable (57.68 ± 8.92 vs. 56.65 ± 8.50, p = 0.614), though symptom frequency and symptom burden subdomains favored the intervention group (p = 0.001 and p = 0.002, respectively) (Table 7; Table 8; Supplementary Table S2; Supplementary Figure S2).

**TABLE 7:** Comparison of clinical and echocardiographic indicators at week 30.

Variable	Intervention (*n* = 111)	Control (*n* = 111)	*t/z*	*p*
Heart rate (bpm)	74.08 ± 12.25	76.38 ± 11.99	0.815	0.418
SBP (mmHg)	122.41 ± 26.04	124.19 ± 29.60	0.275	0.784
DBP (mmHg)	76.97 ± 17.53	75.41 ± 17.18	0.388	0.699
LVEF (%)	55.12% ± 4.85%	56.78% ± 5.42%	1.024	0.312
LVEDD (mm)	66.08 ± 26.83	61.26 ± 15.58	0.945	0.348
LVESD (mm)	53.10 ± 21.80	46.62 ± 11.00	1.615	0.111
LAVI (mL/m^2^)	59.2 ± 20.1	60.5 ± 18.6	0.320	0.750
RATD (mm)	37.16 ± 8.24	38.19 ± 6.96	0.579	0.564
LVEDV (mL)	182.06[136.00,218.00]	138.87[113.00,208.52]	1.633	0.103
LVEDVi (mL/m^2^)	105.2 [78.6, 126.0]	80.3 [65.3, 120.5]	1.122	0.197
LVESV (mL)	107.52[87.22,160.69]	86.75[54.00,138.31]	1.843	0.065
IVST (mm)	22.30 ± 1.56	22.58 ± 1.53	0.780	0.438
LVOT (mm)	12.52 ± 6.81	12.40 ± 0.55	0.108	0.915
LVOT gradient (resting) (mmHg)	23.76 ± 4.09	78.49 ± 13.21	24.074	<0.001**
LVOT gradient (provoked) (mmHg)	48.95 ± 11.10	113.59 ± 14.92	21.147	<0.001**
pvo2	30.65 ± 4.34	28.03 ± 10.46	1.408	0.166
Hs-cTnI	6.50 ± 1.41	13.19 ± 3.09	11.978	<0.001**
KCCQ-23CSS	57.68 ± 8.92	56.65 ± 8.50	0.507	0.614
Tapse	19.49 ± 7.77	16.19 ± 5.29	2.135	0.036*
E/e	17.65 ± 4.87	16.49 ± 4.66	1.049	0.298
ST2	22.47 ± 10.10	20.97 ± 7.39	0.728	0.469
GAL-3	19.71 ± 7.58	16.82 ± 6.97	1.711	0.091
NT-proBNP (pg/mL)	1,082.00[661.00,4211.00]	2,361.00[1,227.00,5059.00]	2.049	0.040*

**p < 0.01, *p < 0.05. Abbreviations: bpm, beats per minute; SBP, systolic blood pressure; DBP, diastolic blood pressure; LVEF, left ventricular ejection fraction; LVEDD, left ventricular end-diastolic diameter; LVESD, left ventricular end-systolic diameter; LAVI, left atrial volume index; RATD, right atrial transverse diameter; LVEDV, left ventricular end-diastolic volume; LVEDVi, left ventricular end-diastolic volume index; LVESV, left ventricular end-systolic volume; IVST, interventricular septal thickness; LVOT, left ventricular outflow tract; pvo_2_, partial pressure of venous oxygen; hs-cTnI, high-sensitivity cardiac troponin I; KCCQ-23CSS, Kansas City Cardiomyopathy Questionnaire-23 clinical summary score; TAPSE, tricuspid annular plane systolic excursion; E/e, ratio of early transmitral flow velocity to early mitral annular tissue velocity; ST2, suppression of tumorigenicity 2; GAL-3, galectin-3; NT-proBNP, N-terminal pro-B-type natriuretic peptide.

**TABLE 8:** KCCQ-23 subdomain scores at Week 30.

Subscale	Intervention group (n = 111)	Control group (n = 111)	t-value	P-value
Symptom frequency	72.45 ± 12.38	65.82 ± 14.56	3.421	0.001^**^
Symptom burden	68.91 ± 13.25	62.47 ± 15.33	3.156	0.002^**^
Physical activity limitation	55.38 ± 16.72	52.19 ± 18.44	1.284	0.201
Quality of life	61.25 ± 14.87	58.63 ± 16.21	1.186	0.237
Social functioning	48.76 ± 19.35	46.52 ± 20.18	0.798	0.426

Data are presented as mean ± standard deviation. **P < 0.01 vs. control group.

### 3.3 Longitudinal trajectory of primary endpoints

LMM revealed significant Group × Time interactions for both resting LVOT gradient (F (3,218) = 412.6, p < 0.001) and provoked LVOT gradient (F (3,218) = 358.4, p < 0.001), indicating divergent temporal trajectories between groups. The intervention group showed continuous reduction from baseline to W30, whereas the control group demonstrated initial increase followed by modest decline, remaining consistently higher than the intervention group at all follow-up time points (Figures 1–4; Supplementary Tables S3, S4).

**FIGURE 1:**
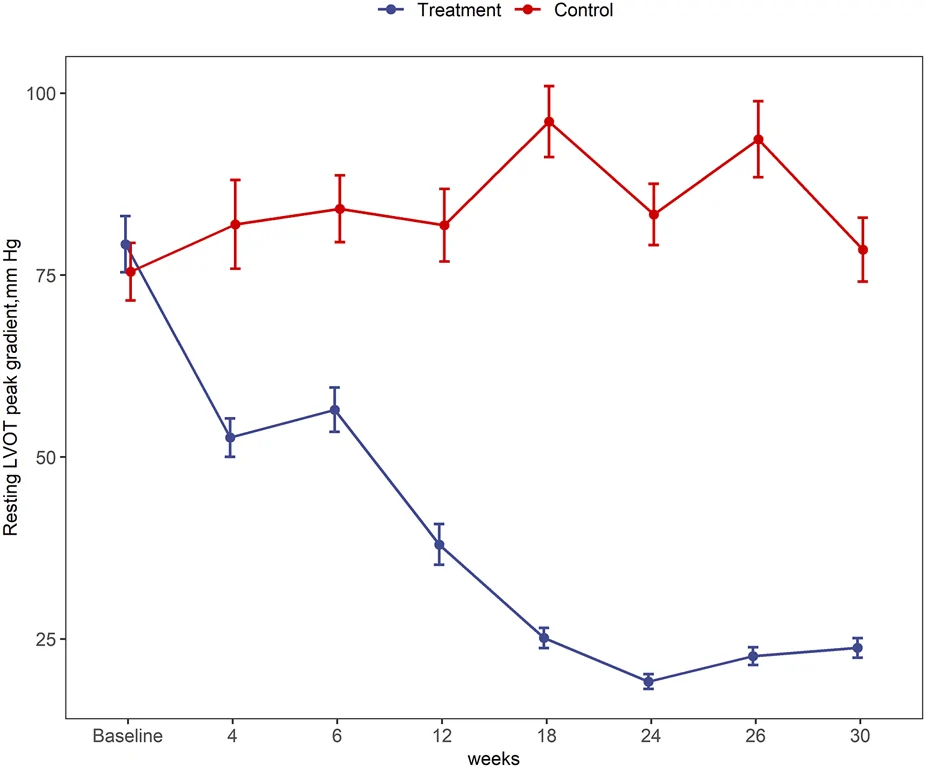
Changes in diastolic LVOT peak gradients over time.

**FIGURE 2:**
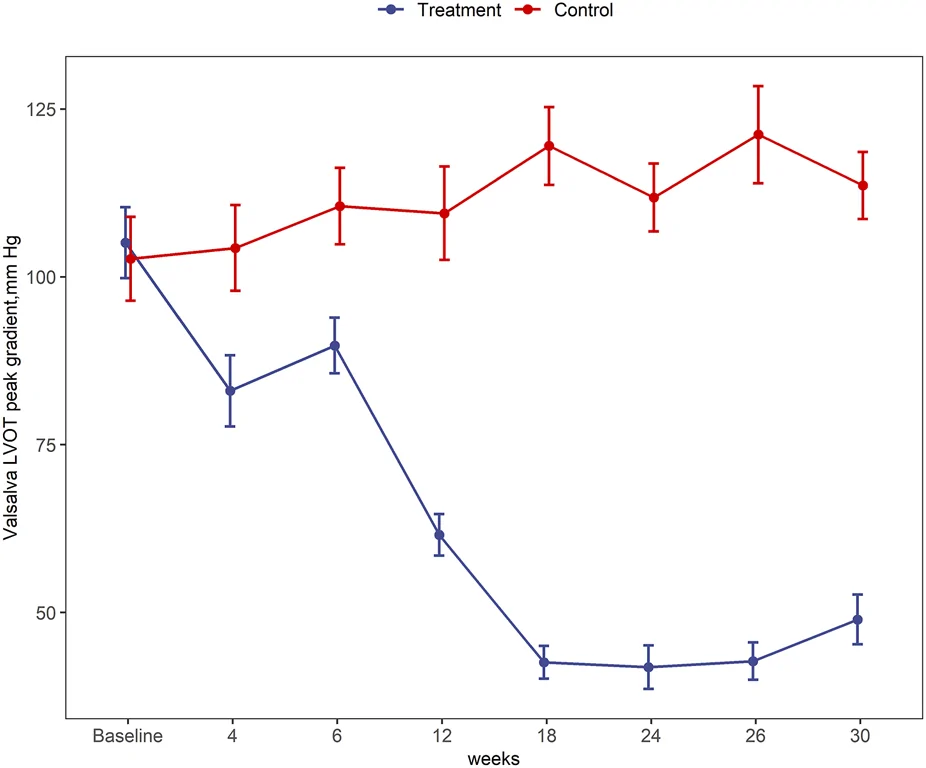
Changes in Valsalva-provoked LVOT peak gradients over time.

**FIGURE 3:**
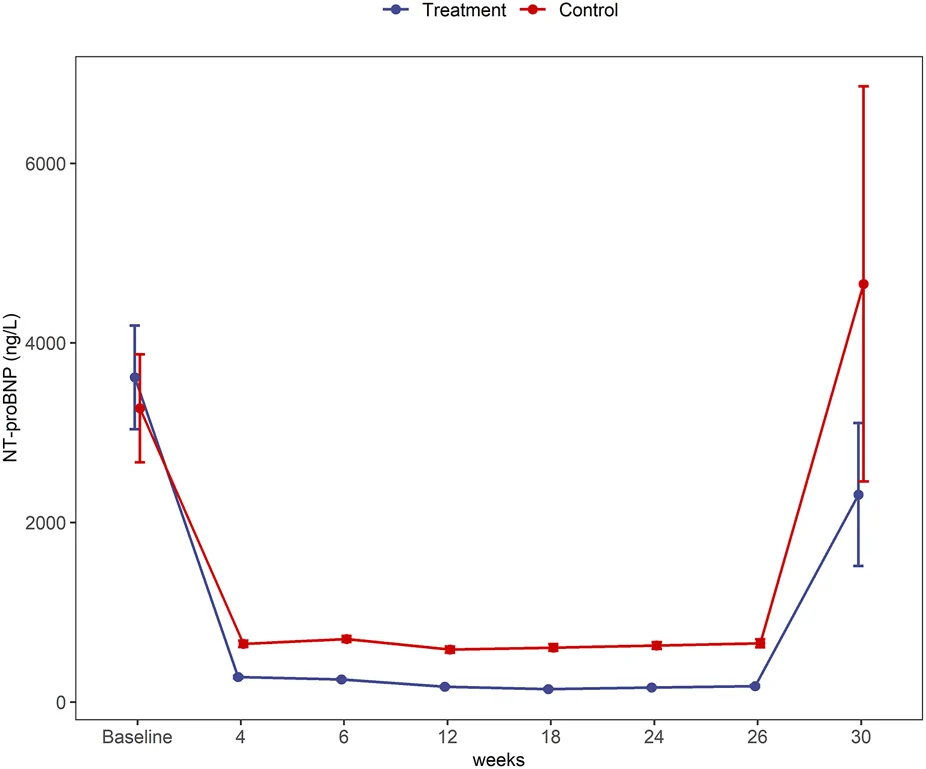
Temporal trends in NT-proBNP levels during follow-up.

**FIGURE 4:**
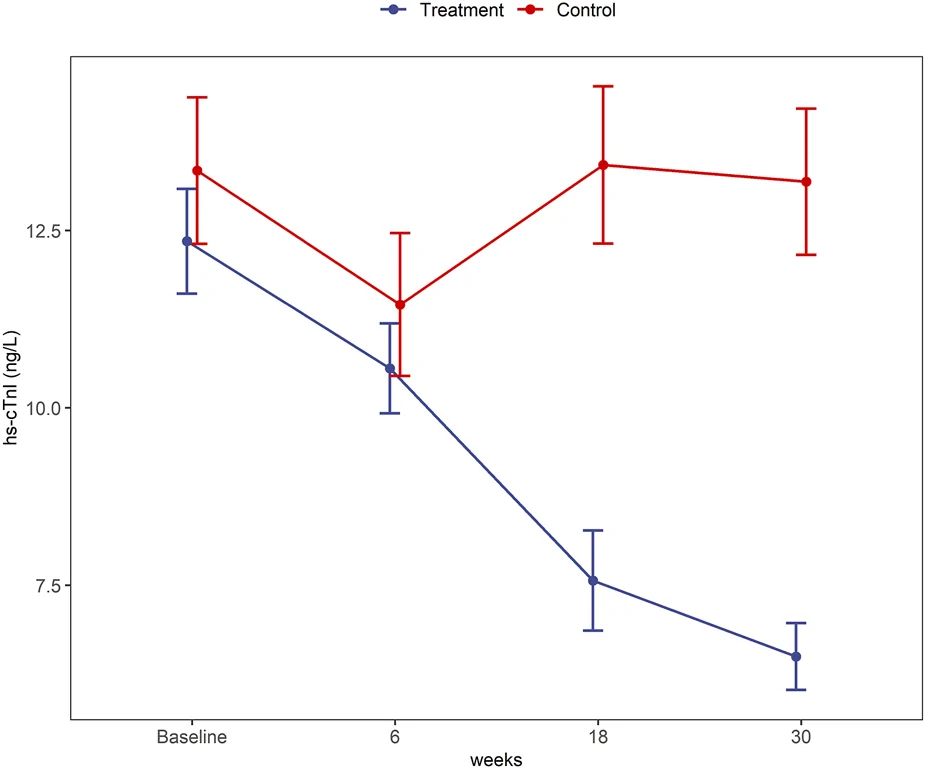
Temporal trends in hs-cTnI levels.

### 3.4 Safety outcomes

LVEF, especially the change in LVEF from baseline (ΔLVEF), was a key indicator for evaluating the safety of mavacamten. We calculated ΔLVEF from baseline (ΔLVEF = [(LVEF_follow-up − LVEF_baseline)/LVEF_baseline] × 100%) at each assessment time point (W6, W12, W18, W24, W30). LVEF differed significantly between the two groups at all assessed time points (p < 0.001), and the greatest intergroup difference was −15.76% at W18. As shown in Table 9; Supplementary Figure S3.

**TABLE 9:** Change in LVEF from baseline (ΔLVEF) over time.

Time point	Intervention group (n = 111)	Control group (n = 111)	t-value	P-value
W6	−8.42% ± 6.35%	−2.15% ± 4.82%	−7.832	<0.001^**^
W12	−12.18% ± 7.64%	−1.89% ± 5.11%	−11.247	<0.001^**^
W18	−15.76% ± 8.92%	−1.52% ± 5.38%	−13.589	<0.001^**^
W24	−14.35% ± 9.15%	−0.98% ± 5.72%	−12.156	<0.001^**^
W30	−13.68% ± 9.47%	−1.25% ± 5.95%	−11.034	<0.001^**^

**P < 0.01 vs. control group at corresponding time point.

LVEF decline to <50% occurred in 12 patients (9.5%) in the intervention group versus 3 (2.2%) in the control group (p = 0.008). Four patients (3.2%) in the intervention group discontinued mavacamten due to LVEF <45%. Overall adverse event rates were comparable between groups (19.0% vs. 20.9%, p = 0.712). Serious adverse events were rare (1.6% vs. 0.7%) (Table 9).

### 3.5 Subgroup analyses

Subgroup analyses examined whether baseline characteristics modified the treatment effect on KCCQ-23CSS change from baseline to Week 30. Linear models with treatment-by-subgroup interaction terms revealed no significant heterogeneity across age, sex, or clinical indicator strata (all p-interaction >0.05; Figure 5; Supplementary Table S5). These findings indicate that the lack of significant overall KCCQ-23CSS improvement with mavacamten was consistent across examined subgroups. Notably, the significant improvements observed in symptom frequency and symptom burden subdomains, coupled with objective 6MWT improvement (Table 10), suggest genuine clinical benefit that may be partially masked by the composite summary score.

**FIGURE 5:**
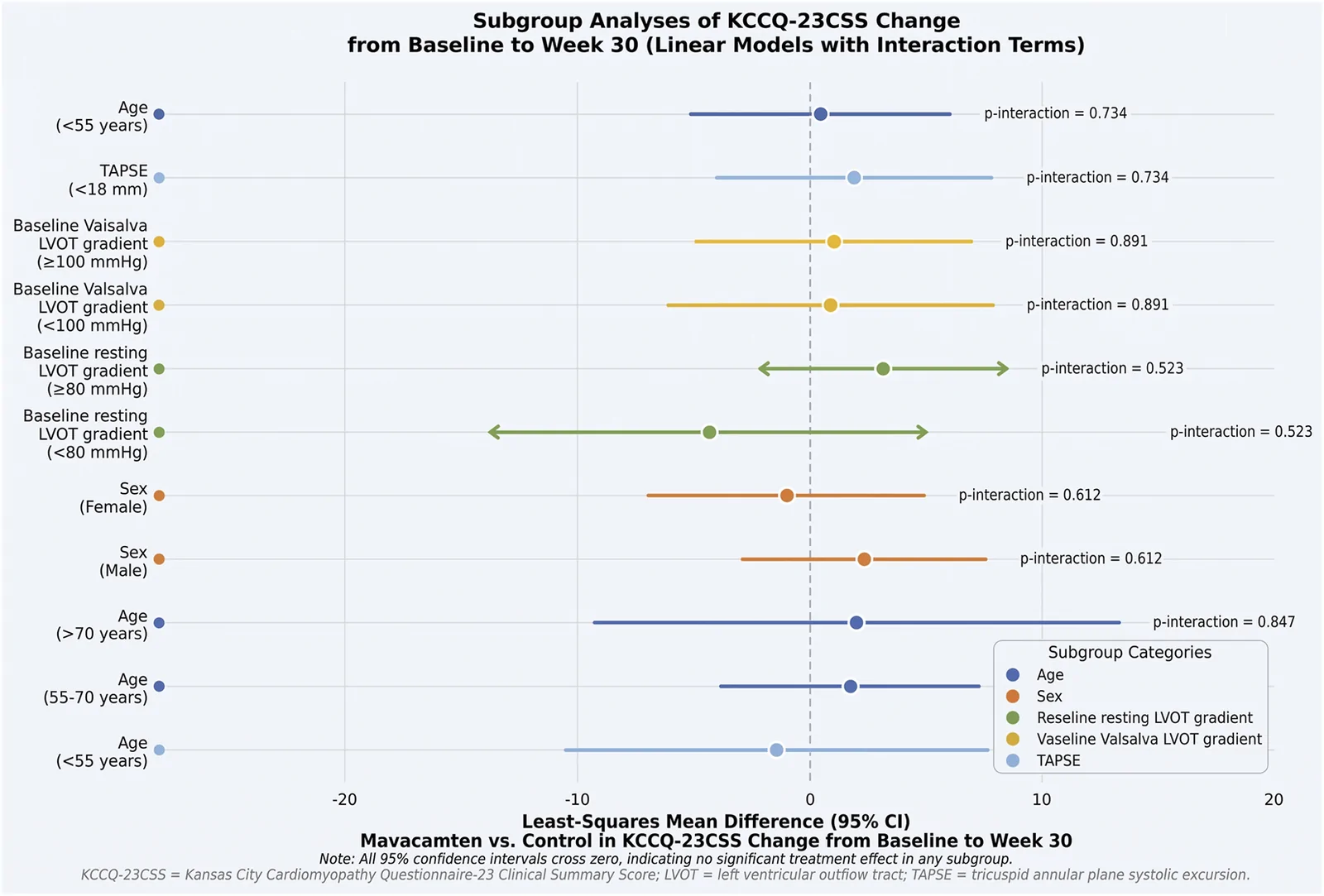
Subgroup analyses of KCCQ-23CSS change from baseline to Week 30 using linear models with interaction terms. LSM = least-squares mean; CI = confidence interval. All 95% confidence intervals cross zero, indicating no significant treatment effect in any subgroup.

**TABLE 10:** Six-minute walk test results over time.

Time point	Intervention group (n = 111)	Control group (n = 111)	t-value	P-value
Baseline	385.42 ± 78.65 m	372.18 ± 82.34 m	1.152	0.251
W6	412.38 ± 75.29 m	378.55 ± 79.47 m	3.089	0.002**
W18	445.67 ± 71.84 m	381.92 ± 76.53 m	6.214	<0.001**
W30	456.83 ± 68.91 m	385.47 ± 74.26 m	7.185	<0.001**

Data are presented as mean ± standard deviation. 6MWT, 6-min walk test. **P < 0.01 vs. control group at corresponding time point.

### 3.6 Concomitant medication use during follow-up

At baseline, background therapy differed between groups. In the intervention group, 89 patients (70.6%) were receiving β-blockers, 31 (24.6%) non-dihydropyridine calcium channel blockers (CCBs), and 6 (4.8%) both agents. In the control group, 102 patients (76.1%) received β-blockers, 28 (20.9%) CCBs, and 4 (3.0%) combination therapy. No patient in either group received disopyramide.

At Week 6, the proportion of patients receiving β-blockers in the intervention group decreased to 62.7% (n = 79), with 18 patients (14.3%) discontinuing β-blockers and 8 (6.3%) reducing dose due to bradycardia or hypotension following mavacamten up-titration. In the control group, β-blocker use increased to 82.1% (n = 110), with dose up-titration in 34 patients (25.4%) to achieve optimal heart rate control. CCB use remained stable in both groups (intervention: 23.8%; control: 22.4%).

At Week 18, 58.7% (n = 74) of intervention-group patients remained on β-blockers, with 21 patients (16.7%) having discontinued background therapy entirely following achievement of LVOT gradient <30 mmHg. In the control group, β-blocker coverage reached 85.1% (n = 114), with 41 patients (30.6%) on maximally tolerated doses.

At Week 30, 55.6% (n = 70) of mavacamten-treated patients continued β-blockers, 22.2% (n = 28) continued CCBs, and 4.0% (n = 5) remained on dual therapy. In the control group, 86.6% (n = 116) were receiving β-blockers, 20.9% (n = 28) CCBs, and 3.0% (n = 4) dual therapy. The mean equivalent daily doses of metoprolol succinate were 95.3 ± 42.6 mg in the intervention group versus 142.8 ± 51.2 mg in the control group at Week 30 (p < 0.001), reflecting differential dose optimization strategies between groups.

## 4 Discussion

Although pharmacological agents such as β-blockers, non-dihydropyridine calcium channel blockers, and disopyramide can alleviate clinical symptoms in individuals with symptomatic obstructive HCM, these therapies do not directly target the underlying pathophysiological mechanisms of the disease (Ommen et al., 2020). Current clinical guidelines recommend myosin inhibitors when symptoms related to LVOT obstruction persist despite treatment with β-blockers or non-dihydropyridine calcium channel blockers (Ommen et al., 2024; National Center for Cardiovascular Diseases, 2023; National Expert Committee on Cardiovascular Diseases, 2022).

In the PIONEER-HCM trial, mavacamten monotherapy reduced Valsalva-provoked LVOT gradient by 89.5 mmHg, with 8 of 11 participants achieving values below 30 mmHg (Heitner et al., 2019). In our study, the intervention group maintained a diastolic LVOT peak gradient of 23.76 ± 4.09 mmHg at week 30, while the control group remained elevated at 78.49 ± 13.21 mmHg (t = 24.074, p < 0.001), consistent with the profound obstruction relief observed with mavacamten monotherapy. Notably, the concomitant use of β-blockers in the lower-dose PIONEER-HCM cohort resulted in a substantially attenuated gradient reduction (25 mmHg), suggesting that concurrent β-blocker therapy may limit the hemodynamic efficacy of mavacamten-a consideration relevant to our real-world dosing protocol.

The differential use and dosing of background negative inotropic therapy between groups may have influenced the observed treatment effects. In the intervention group, the progressive reduction in β-blocker use and dosing-from 70.6% at baseline to 55.6% at Week 30-likely reflects physicians' response to the combined negative inotropic effects of mavacamten and background therapy. This de-escalation may have partially offset the full hemodynamic potential of mavacamten, as suggested by the PIONEER-HCM trial, where concomitant β-blocker use attenuated the LVOT gradient reduction from 89.5 mmHg to 25 mmHg (Heitner et al., 2019). Conversely, in the control group, the intensification of β-blocker therapy (from 76.1% to 86.6%) and higher mean equivalent doses at Week 30 represent a treat-to-target approach that may have narrowed the apparent efficacy gap between conventional therapy and mavacamten. This phenomenon-where control-group patients receive optimized standard care while intervention-group patients de-escalate background therapy due to perceived adequacy of mavacamten-introduces a form of performance bias that may underestimate the true incremental benefit of mavacamten over conventional pharmacotherapy. Furthermore, the absence of disopyramide use at our institution, while reflecting real-world Chinese practice, means that our control group did not represent the maximal pharmacological armamentarium available in Western centers, potentially limiting the external validity of our comparative efficacy estimates. Future studies should systematically report and adjust for temporal changes in background medication use to isolate the independent effect of mavacamten on hemodynamic and clinical outcomes.

In MAVERICK-HCM, mavacamten reduced NT-proBNP by 53% and cTnI by 34% in patients with non-obstructive HCM, yet no significant benefit was observed in KCCQ-23CSS (Ho et al., 2020). By contrast, our study in obstructive HCM demonstrated significant improvements in symptom frequency and burden subdomains, alongside objective 6MWT improvement. The lack of overall KCCQ-23CSS difference in our cohort-despite clear hemodynamic improvement-likely reflects higher baseline scores (55–58 vs. ∼45 in EXPLORER-HCM), suggesting a milder symptomatic burden with reduced ceiling for improvement. Additionally, the Chinese language version of KCCQ-23 may incompletely capture exertional dyspnea specific to Chinese patients with oHCM, and the 30-week follow-up may be insufficient for symptomatic benefits to fully manifest after objective hemodynamic improvement.

In EXPLORER-HCM, mavacamten achieved an 80.9% resolution rate of mitral valve systolic anterior motion and significant improvements in LAVI and lateral wall E/e′, with reductions in LAVI correlated with increased peak oxygen consumption (Olivotto et al., 2020). Our echocardiographic findings at weeks 6, 18, and 30 align with these observations, demonstrating sustained low resting and Valsalva LVOT gradients in the intervention group. The stable heart rate throughout follow-up in our cohort further supports a primary pharmacodynamic effect of mavacamten on myocardial contractility rather than a chronotropic mechanism, consistent with the EXPLORER-HCM pharmacodynamic profile.

Tian et al. reported that mavacamten significantly improved LVOT gradients and all secondary endpoints in Chinese patients with HCM, with good tolerability and no new safety signals (Tian et al., 2023). Garcia-Pavia et al. reported that sustained treatment with mavacamten for up to 120 weeks was associated with continuous improvement in LVOT obstruction, related clinical symptoms, and NT-proBNP levels (Garcia-Pavia et al., 2023). Desai et al. showed that 56 weeks of mavacamten treatment reduced the need for surgical intervention while sustaining improvements in LVOT gradients and clinical symptoms (Desai et al., 2023); our results support this therapeutic potential, though close monitoring for left ventricular systolic dysfunction remains essential. More recently, Masri et al. reported interim results from the PIONEER-OLE study demonstrating that mavacamten maintained sustained reductions in resting (−50 mmHg) and Valsalva (−70 mmHg) LVOT gradients, alongside NYHA class improvements and NT-proBNP reduction, over a median follow-up of 201 weeks with predominantly mild-to-moderate adverse events (Masri et al., 2024). Our 30-week findings are consistent with these reports in demonstrating durable hemodynamic association with improved outcomes; however, the extended follow-up in PIONEER-OLE underscores the need for longer observation in our cohort to confirm sustained benefit and monitor for late-onset LVEF reduction, which occurred in one patient in that study and recovered with dose adjustment. There was no statistically significant between-group difference in LVEF at Week 30 (55.12% vs. 56.78%, p = 0.312), indicating overall LVEF stabilization within a clinically acceptable range for most mavacamten recipients. Nevertheless, a 2.7% rate of LVEF <45% reinforces the importance of routine echocardiographic monitoring and standardized dose adjustment protocols. With mandatory echocardiographic surveillance and appropriate dose modification or discontinuation for meaningful LVEF decline, the overall risk–benefit profile remains favorable for mavacamten therapy.

Subgroup analyses from EXPLORER-HCM indicated that female patients, despite greater baseline disease burden (lower peak oxygen consumption (pVO_2_), higher NYHA class III proportion, higher NT-proBNP, lower KCCQ-23CCS), experienced comparable composite endpoint improvements with twofold greater reductions in KCCQ-23CCS and NT-proBNP than male patients (Cresci et al., 2024). Our study did not stratify by sex, but these findings suggest a potentially greater therapeutic benefit of mavacamten in female patients that warrants investigation in Chinese cohorts. Giudicessi et al. reported that mavacamten may confer greater benefits in younger, gene-positive patients with oHCM (Giudicessi et al., 2024); however, the small sample size and limited NYHA class II inclusion restrict generalizability.

Our results indicate that mavacamten inhibits cardiac myosin activity, thereby improving systolic function, reducing dynamic LVOT obstruction, and improving biomarkers associated with cardiac filling pressure and pressure load (Maron et al., 2024; Bishev et al., 2023; Alharbi et al., 2024). The sustained low resting LVOT gradient maintained in our intervention group over 30 weeks, alongside significant Valsalva gradient reduction (48.95 ± 11.10 mmHg vs. 113.59 ± 14.92 mmHg in controls; t = 21.147, p < 0.001), confirms durable hemodynamic association with improved outcomes.

Complementing these functional improvements, cardiac magnetic resonance (CMR) substudy analyses of the EXPLORER-HCM trial have demonstrated that mavacamten favorably impacts cardiac structure, reducing left ventricular mass and providing evidence of reverse remodeling beyond hemodynamic relief (Saberi et al., 2021). A recent 1-year comprehensive CMR study further confirmed sustained functional and structural improvements, including favorable left atrial volume regression and reductions in global myocardial work indices (Seuthe et al., 2025). Although our study did not incorporate serial CMR imaging, the echocardiographic improvements in LAVI and diastolic function parameters observed in our cohort are consistent with these CMR-documented structural remodeling benefits. These findings collectively support the paradigm of mavacamten as a disease-modifying therapy that addresses both the functional and structural manifestations of oHCM.

## 5 Study limitations

This study was limited by its retrospective design and relatively small sample size. Patient grouping was determined based on the clinical availability of mavacamten following its official approval on 24 April 2024, standardized diagnostic and therapeutic eligibility criteria, and comprehensive clinical physician judgment. These inherent retrospective features may introduce potential selection bias and confounding factors, thereby restricting the statistical robustness and generalizability of the present findings. We identified significant baseline imbalances in LVEDD, LAVI, and LVESV between treatment groups, reflecting the non-randomized nature of treatment assignment. While multivariable adjustment and propensity score methods attenuated the observed treatment effects-particularly for NT-proBNP, where the effect became marginally non-significant (P = 0.098) after full adjustment-the primary hemodynamic endpoints (LVOT gradients) remained robustly significant. This pattern suggests that baseline structural differences partially confounded biomarker outcomes but did not fully account for the profound obstruction relief observed with mavacamten. Nevertheless, we cannot exclude residual confounding from unmeasured variables, including myocardial fibrosis burden, specific sarcomere genetic variants, or prior treatment intensity and duration, and our findings should therefore be interpreted as associative rather than causal.

The 30-week follow-up duration represents an important limitation. Although this observation period was sufficient to demonstrate early hemodynamic improvements, it precludes definitive conclusions regarding the long-term durability of these benefits and the sustained safety of mavacamten therapy. Specifically, the delayed onset of left ventricular systolic dysfunction-a recognized risk with myosin inhibition—cannot be adequately evaluated within this timeframe, as prior extension studies have observed reversible reductions in LVEF during prolonged treatment. Furthermore, symptomatic improvements may lag behind objective hemodynamic changes; the lack of significant overall KCCQ-23CSS improvement in our cohort may partly reflect insufficient follow-up duration for patients to fully perceive and report functional gains after LVOT gradient reduction. The impact of mavacamten on long-term clinical outcomes, including the cumulative need for septal reduction therapy, cardiovascular hospitalization, and mortality, also remains unknown. Additionally, the single-center design limits generalizability to broader, multiethnic populations and may reflect institution-specific treatment practices or referral patterns. Future prospective, multicenter studies with extended follow-up, standardized genotyping, and comprehensive cardiac imaging are warranted to validate our findings and establish the long-term efficacy and safety profile of mavacamten in this patient population.

## 6 Conclusion

This study suggests that mavacamten use is associated with clinically meaningful improvements in key cardiac function indicators in patients with oHCM, including LVOT gradient and biomarkers. However, as a retrospective observational study with physician-driven, non-randomized treatment allocation, these associations do not establish causality. The control group received conventional negative-inotrope therapy, which differs mechanistically from mavacamten’s selective cardiac myosin inhibition, introducing channeling bias and confounding by indication. Neither CYP2C19 metabolizer phenotyping nor systematic HCM genetic testing was performed, precluding personalized treatment stratification and mechanistic insights. Definitive conclusions regarding treatment efficacy await prospective randomized controlled trials incorporating pharmacogenomic and genetic analyses.

## Data Availability

The raw data supporting the conclusions of this article will be made available by the authors, without undue reservation.
