# Supplementary material for: Clinical effectiveness of mavacamten on cardiac function and disease-related indicators in patients with obstructive hypertrophic cardiomyopathy
Source: Front Pharmacol. 2026 Oct 27;17:1861248. doi: 10.3389/fphar.2026.1861248 (PMC13747769; doi:10.3389/fphar.2026.1861248)
Supplement: Supplementary file 1 [file Table1.docx]

| Endpoint | Time Point | Intervention Group | Control Group | t/z statistic | p-value |
| --- | --- | --- | --- | --- | --- |
| **Resting LVOT peak gradient (mmHg)** | Baseline | 79.24 ± 11.61 | 75.46 ± 11.86 | 1.387 | 0.17 |
|  | W6 | 56.49 ± 9.15 | 84.11 ± 13.70 | 10.201 | <0.001 |
|  | W18 | 25.14 ± 4.16 | 96.08 ± 14.62 | 28.385 | <0.001 |
|  | W30 | 23.76 ± 4.09 | 78.49 ± 13.21 | 24.074 | <0.001 |
| **Valsalva LVOT peak gradient (mmHg)** | Baseline | 105.08 ± 15.90 | 102.68 ± 18.76 | 0.595 | 0.554 |
|  | W6 | 89.76 ± 12.45 | 110.51 ± 17.04 | 5.981 | <0.001 |
|  | W18 | 42.57 ± 7.34 | 119.49 ± 17.33 | 24.867 | <0.001 |
|  | W30 | 48.95 ± 11.10 | 113.59 ± 14.92 | 21.147 | <0.001 |
| **hs-cTnI (ng/L)** | Baseline | 12.35 ± 2.21 | 13.34 ± 3.10 | 1.593 | 0.116 |
|  | W6 | 10.56 ± 1.90 | 11.46 ± 3.02 | 1.534 | 0.13 |
|  | W18 | 7.57 ± 2.12 | 13.42 ± 3.32 | 9.042 | <0.001 |
|  | W30 | 6.50 ± 1.41 | 13.19 ± 3.09 | 11.978 | <0.001 |
| **NT-proBNP (ng/L)** | Baseline | 3614.79 ± 1734.02 | 3269.70 ± 1801.91 | 0.839 | 0.404 |
|  | W6 | 250.35 ± 31.32 | 701.41 ± 100.46 | 26.073 | <0.001 |
|  | W18 | 143.35 ± 26.58 | 606.43 ± 113.20 | 24.224 | <0.001 |
|  | W30 | 2309.35 ± 2386.76 | 4656.95 ± 6601.02 | 2.034 | 0.048 |

Supplementary Table S1. Unadjusted clinical outcomes at each follow-up time point
