# Supplementary material for: Clinical effectiveness of mavacamten on cardiac function and disease-related indicators in patients with obstructive hypertrophic cardiomyopathy
Source: Front Pharmacol. 2026 Oct 27;17:1861248. doi: 10.3389/fphar.2026.1861248 (PMC13747769; doi:10.3389/fphar.2026.1861248)
Supplement: Supplementary file 2 [file Table2.docx]

| Time Point | Intervention Group | Control Group | p-value |
| --- | --- | --- | --- |
| Baseline | 58.42 ± 2.89% | 58.38 ± 2.68% | 0.626 |
| W6 | 57.15 ± 3.12% | 58.01 ± 2.95% | 0.038 |
| W12 | 56.28 ± 3.45% | 57.85 ± 3.10% | 0.002 |
| W18 | 55.68 ± 4.02% | 57.42 ± 3.88% | 0.003 |
| W24 | 55.35 ± 4.56% | 57.10 ± 4.25% | 0.005 |
| W30 | 55.12 ± 4.85% | 56.78 ± 5.42% | 0.312 |

Supplementary Table 2 Left ventricular ejection fraction trajectory over time
