# Supplementary material for: Clinical effectiveness of mavacamten on cardiac function and disease-related indicators in patients with obstructive hypertrophic cardiomyopathy
Source: Front Pharmacol. 2026 Oct 27;17:1861248. doi: 10.3389/fphar.2026.1861248 (PMC13747769; doi:10.3389/fphar.2026.1861248)
Supplement: Supplementary file 3 [file Table3.docx]

Supplementary Table 3. Comparison of clinical and echocardiographic indicators across all time points

| Variable | Intervention | Control | *t* | *p* |
| --- | --- | --- | --- | --- |
| Baseline | - | - | - | - |
| Resting LVOT peak gradient (mmHg) | 79.24±11.61 | 75.46±11.86 | 1.387 | 0.170 |
| Valsalva LVOT peak gradient (mmHg) | 105.08±15.90 | 102.68±18.76 | 0.595 | 0.554 |
| hs-cTnI (ng/L) | 12.35±2.21 | 13.34±3.10 | 1.593 | 0.116 |
| NT-proBNP (ng/L) | 3614.79±1734.02 | 3269.70±1801.91 | 0.839 | 0.404 |
| W6 | - | - | - | - |
| Resting LVOT peak gradient (mmHg) | 56.49±9.15 | 84.11±13.70 | 10.201 | <0.001** |
| Valsalva LVOT peak gradient (mmHg) | 89.76±12.45 | 110.51±17.04 | 5.981 | <0.001** |
| hs-cTnI (ng/L) | 10.56±1.90 | 11.46±3.02 | 1.534 | 0.130 |
| NT-proBNP (ng/L) | 250.35±31.32 | 701.41±100.46 | 26.073 | <0.001** |
| W18 | - | - | - | - |
| Resting LVOT peak gradient (mmHg) | 25.14±4.16 | 96.08±14.62 | 28.385 | <0.001** |
| Valsalva LVOT peak gradient (mmHg) | 42.57±7.34 | 119.49±17.33 | 24.867 | <0.001** |
| hs-cTnI (ng/L) | 7.57±2.12 | 13.42±3.32 | 9.042 | <0.001** |
| NT-proBNP (ng/L) | 143.35±26.58 | 606.43±113.20 | 24.224 | <0.001** |
| W30 | - | - | - | - |
| Resting LVOT peak gradient (mmHg) | 23.76±4.09 | 78.49±13.21 | 24.074 | <0.001** |
| Valsalva LVOT peak gradient (mmHg) | 48.95±11.10 | 113.59±14.92 | 21.147 | <0.001** |
| hs-cTnI (ng/L) | 6.50±1.41 | 13.19±3.09 | 11.978 | <0.001** |
| NT-proBNP (pg/mL) | 2309.35±2386.76 | 4656.95±6601.02 | 2.034 | 0.048* |

Note: ***p*<0.01, **p*<0.05. Abbreviations: LVOT, left ventricular outflow tract; hs-cTnI, high-sensitivity cardiac troponin I; NT-proBNP, N-terminal pro-B-type natriuretic peptide.
