# Supplementary material for: Clinical effectiveness of mavacamten on cardiac function and disease-related indicators in patients with obstructive hypertrophic cardiomyopathy
Source: Front Pharmacol. 2026 Oct 27;17:1861248. doi: 10.3389/fphar.2026.1861248 (PMC13747769; doi:10.3389/fphar.2026.1861248)
Supplement: Supplementary file 4 [file Table4.docx]

Supplementary Table 4 Matched outcomes at Week 30

| Outcome | Matched Intervention | Matched Control | p-value |
| --- | --- | --- | --- |
| Resting LVOT gradient at W30 | 23.91 ± 4.22 mmHg | 78.33 ± 13.05 mmHg | <0.001 |
| Provoked LVOT gradient at W30 | 49.12 ± 11.25 mmHg | 113.28 ± 14.76 mmHg | <0.001 |
