# Supplementary material for: Clinical effectiveness of mavacamten on cardiac function and disease-related indicators in patients with obstructive hypertrophic cardiomyopathy
Source: Front Pharmacol. 2026 Oct 27;17:1861248. doi: 10.3389/fphar.2026.1861248 (PMC13747769; doi:10.3389/fphar.2026.1861248)
Supplement: Supplementary file 5 [file Table5.docx]

Supplementary Table 5 Sensitivity Analyses for the Treatment Effect on KCCQ-23 Clinical Summary Score

| Analysis Method | Intervention Group | Control Group | Between-Group Difference (95% CI) | P-value |
| --- | --- | --- | --- | --- |
|  | Mean ± SD (mmHg) | Mean ± SD (mmHg) | |  |
| **Unadjusted analysis** | 23.76 ± 4.09 | 78.49 ± 13.21 | −54.73 (−57.89 to −51.57) | <0.001 |
| **Propensity score matching (1:1)** | 24.15 ± 4.38 | 77.82 ± 12.95 | −53.67 (−56.98 to −50.36) | <0.001 |
| **Inverse probability of treatment weighting (IPTW)** | 23.98 ± 4.21 | 78.15 ± 13.08 | −54.17 (−57.31 to −51.03) | <0.001 |
| **Covariate-adjusted regression** | 24.32 ± 4.55 | 77.65 ± 12.87 | −53.33 (−56.72 to −49.94) | <0.001 |
| **E-value analysis** | — | — | Lower bound: 2.41 | — |
| **Negative control outcome (right atrial diameter)** | 34.92 ± 3.83 | 34.97 ± 3.44 | −0.05 (−1.12 to 1.02) | 0.926 |
